# Two new species of the *Phanaeus endymion* species group (Coleoptera, Scarabaeidae, Scarabaeinae)

**DOI:** 10.3897/zookeys.702.14728

**Published:** 2017-09-26

**Authors:** Victor Moctezuma, José Luis Sánchez-Huerta, Gonzalo Halffter

**Affiliations:** 1 Instituto de Ecología, A.C., Carretera antigua a Coatepec 351, El Haya, Xalapa, Veracruz, 91070, Mexico

**Keywords:** Dung beetle, Mexican Transition Zone, mycophagy, Phanaeini, Sierra Madre del Sur, Trans-Mexican Volcanic Belt, Micofagia, escarabajos del estiércol, Phanaeini, Sierra Madre del Sur, Sistema Volcánico Transversal, Zona de Transición Mexicana

## Abstract

*Phanaeus bravoensis* **sp. n.** is described from the coniferous-oak forests in the state of Guerrero, and *P. huichol* **sp. n.** from coniferous-oak forests and cloud forests in Jalisco and Nayarit. The new species are closely related to *P. halffterorum* and *P. zoque* respectively. Morphological trait combination, geographic distribution, and trophic habits show important differences among the studied species. A distribution map and an updated key to separate the species are included.

## Abstract (es)

Describimos a *Phanaeus bravoensis* **sp. n.** proveniente de los bosques de coníferas y encino del estado de Guerrero, y a *P. huichol* **sp. n.** de los bosques de coníferas y encino y bosques de niebla de Jalisco y Nayarit. Las nuevas especies están cercanamente relacionadas con *P. halffterorum* y *P. zoque*, respectivamente. Las combinaciones de caracteres morfológicos, áreas de distribución geográfica y los hábitos alimenticios muestran diferencias importantes entre las especies estudiadas. Incluimos un mapa de distribución y una clave actualizada para separar a las especies.

## Introduction

*Phanaeus* Macleay, 1819 is a new world genus of dung beetles that presents a bright metallic coloration and a pronounced sexual dimorphism (males with large cephalic horns and striking pronotal projections) (Edmonds 2006). *Phanaeus* includes at least 55 valid species and 12 species groups. Several studies of *Phanaeus* have been published in recent years, including major reviews (Edmonds 1994, Arnaud 2002, Edmonds and Zídek 2012). As a consequence, there is a broad knowledge of the geographical distribution of *Phanaeus*, and it is considered a Neotropical taxon of South American origin. The subgenerus *Phanaeus* s. str. probably colonized the Mexican Transition Zone during the Miocene, where it diversified and expanded northward into the U.S.A. The subgenus Notiophanaeus, however, radiated and expanded in South America and one species group arrived to the Mexican Transition Zone prior to closure of the Panama land bridge: the *endymion* species group (Halffter and Morrone 2017).

The *Phanaeus endymion* species group brings together five closely related Mesoamerican species: *Phanaeus endymion* Harold, 1863, *P. halffterorum* Edmonds, 1979, *P. pyrois* Bates, 1887, *P. zapotecus* Edmonds, 2006 and *P. zoque* Moctezuma & Halffter, 2017 (Moctezuma and Halffter 2017), in addition to the two new species described here. *Phanaeus halffterorum* was described with 17 individuals from Estado de Mexico and one male from Guerrero. Edmonds (1979) included among the features of *P. halffterorum* a strong acute tooth in the middle of anterior margin of pronotum (a character present even in the smallest individuals), with the exception of the Guerrero specimen. We studied the *P. halffterorum* type series and new specimens collected in Guerrero, and we have concluded that the *halffterorum* population from Guerrero represents a new species, which is described in this work. *Phanaeus endymion* is a tropical species that appeared to present a disjunct population in temperate forests from the Mexican Pacific slope of the Trans-Mexican Volcanic Belt (Edmonds 1994, Edmonds and Zídek 2012). In this study, we also recognize these “*endymion*” Pacific slope populations as a new species, which is closely related to *P. zoque*.

## Methods

The studied specimens are deposited in the following collections:

**CEMT** Seção de Entomologia da Coleção Zoológica, Departamento de Biologia e Zoologia, Universidade Federal de Mato Grosso, Cuiabá, MG, BRA

**CNIN** Colección Nacional de Insectos, Instituto de Biología, Universidad Nacional Autónoma de México, México City, MX

**IEXA** Colección Entomológica, Instituto de Ecología, A. C., Xalapa, Ver., MX

**TAMU** Texas A&M University Insect Collection, TX, USA

**FSCA** Florida State Collection of Arthropods, Gainesville, FL, USA

**CDINECOL** C Deloya Collection - Instituto de Ecología, A. C., Xalapa, Ver., MX

**JLSHC** JL Sánchez-Huerta Collection, Xalapa, Veracruz, MX

**MXAL** MA Morón Collection, Xalapa, Veracruz, MX

**VMC** V Moctezuma Collection, Xalapa, Veracruz, MX

For this study, the phylogenetic species concept is used (Wheeler and Platnick 2000), which defines species as the smallest aggregation of populations diagnosable by a unique combination of character states. Type specimens bear determination labels printed with black ink on acid-free red paper. The aedeagus and internal sac were prepared as outlined by Marchisio and Zunino (2012) and Moctezuma and Halffter (2017). All measurements and pictures (z-stack image capture method) were taken using a Leica Z16APOA stereomicroscope and the fabricant software.

## Taxonomy

### Phanaeus halffterorum

Taxon classificationAnimaliaColeopteraScarabaeidae

Edmonds, 1979

Figs 1 , 2 , 3 , 4

Phanaeus halffterorum : Edmonds (1979: 99; *partim*), Halffter and Edmonds (1982: 88–89), Anduaga and Halffter (1991: 157), Delgado-Castillo et al. (1993: 125), Edmonds (1994: 39–43, 101), Anduaga (2000: 125, 130), Arnaud (2002: 95–96), Edmonds (2003: 61, 65), Edmonds (2006: 31, 34, 36), Ceballos et al. (2009: 397), Edmonds and Zídek (2012: 3, 5, 12, 52, 54), Deloya et al. (2014: 77), Moctezuma and Halffter (2017: 52, 54–55), Lizardo et al. (in press).

#### Type material examined

(5 ♂♂, 2 ♀♀). Paratypes (TAMU): 2 ♂♂, 2 ♀♀ labeled “MEXICO: Mexico, 5 km E Temascaltepec, Real de Arriba, 2200 m, 10-VII-1976, Fungus, oak-pine forest, W.D. Edmonds, P. Reyes, B. Kohlmann cols.”; 2 ♂♂ labeled “MEXICO: Mexico, 8 km W Temascaltepec, 2360 m, 11-VII-76, Fungus in pine-oak forest, W.D. Edmonds, P. Reyes and B. Kohlmann cols.”; 1 ♂ labeled “Real de Arriba, Dist. Temascaltepec, Edo. Mex., VII-1932, 6300 ft, Mexico D.F., Hinton Coll. B.M.1939-583”.

**Figure 1.**
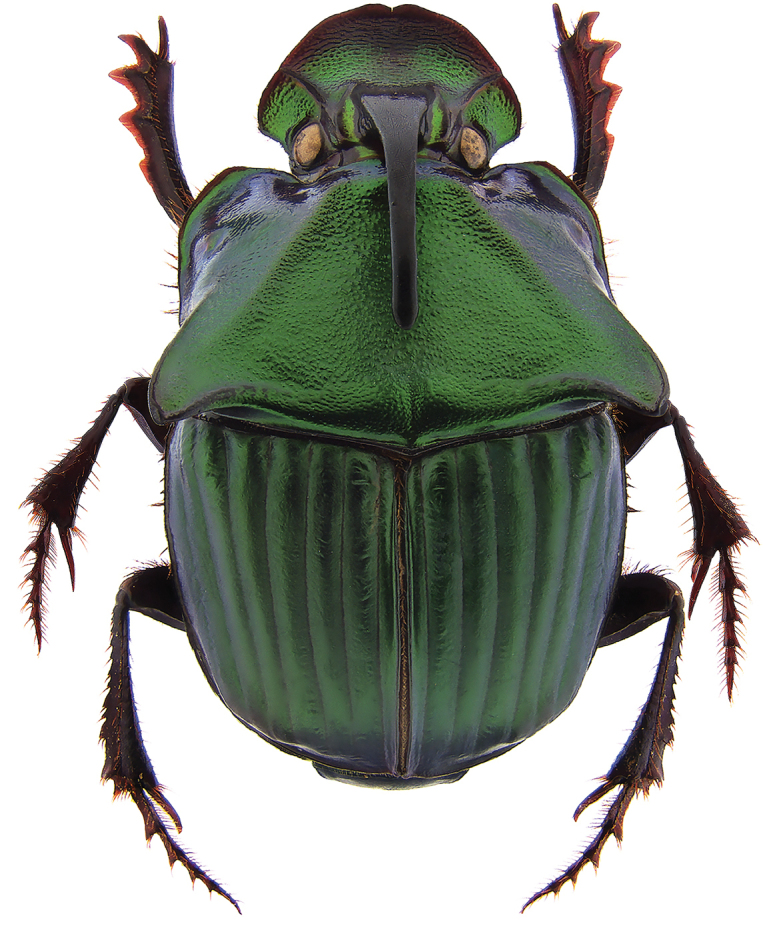
*Phanaeus halffterorum* major male, dorsal view (paratype).

**Figure 2.**
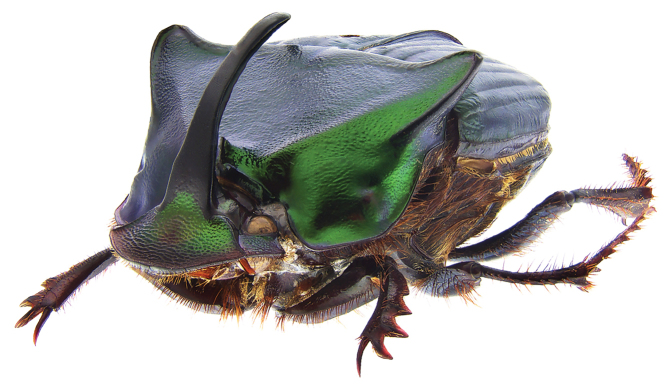
*Phanaeus halffterorum* major male, lateral view (paratype).

**Figure 3.**
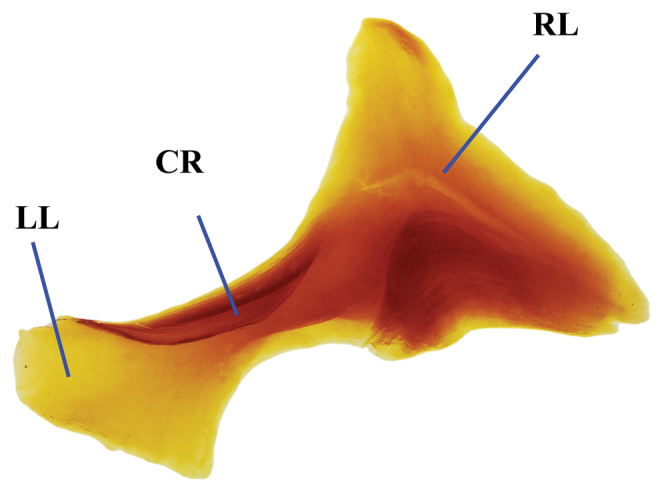
Lamella copulatrix of *Phanaeus halffterorum* (paratype). Abbreviations: LL: left lobe, CR: central ridge, RL: right lobe.

**Figure 4.**
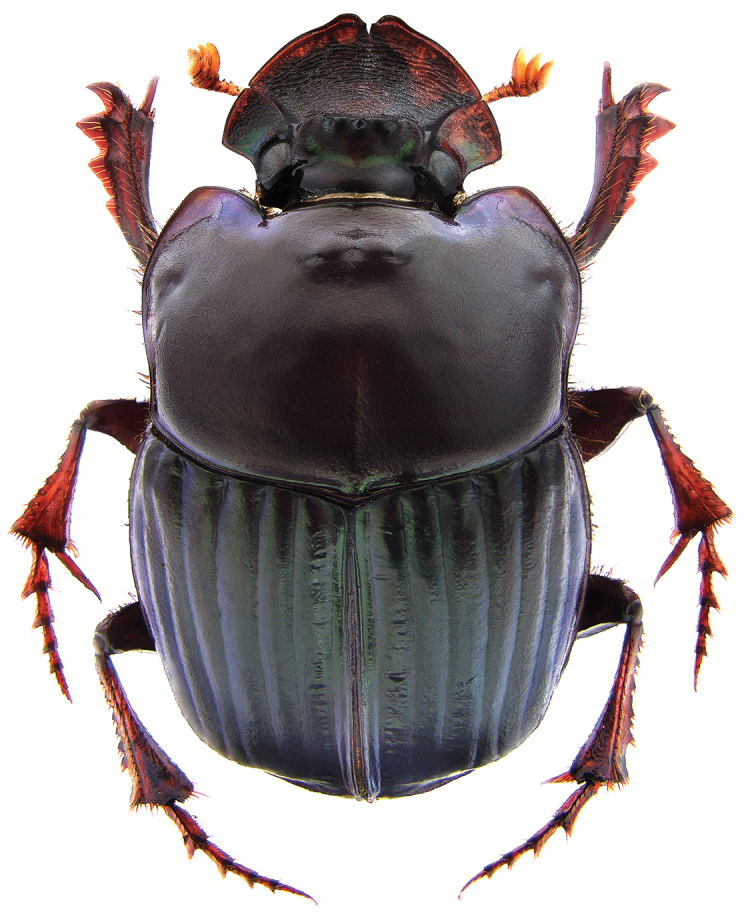
*Phanaeus halffterorum* female, dorsal view (paratype).

#### Distribution and ecology.

**Figure 5.**
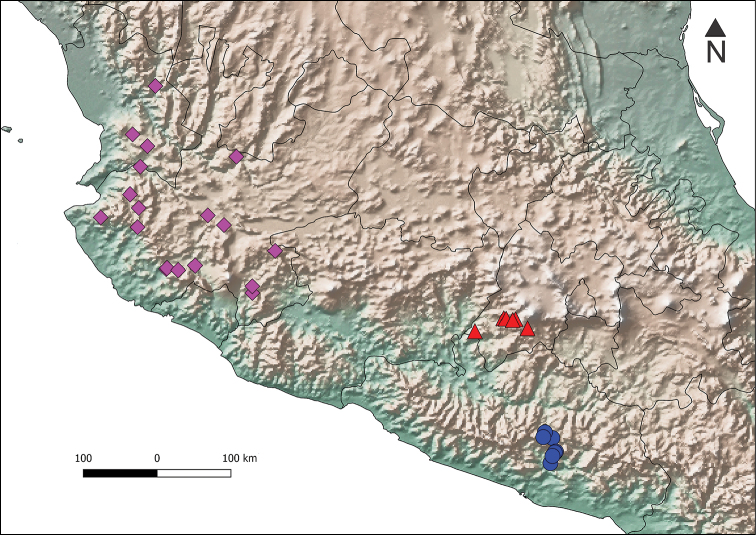
Distribution of *Phanaeus halffterorum* (red triangle), *P. bravoensis* sp. n. (blue circle) and *P. huichol* sp. n. (purple diamonds).

This species is known from the environs of Temascaltepec, State of Mexico (Fig. 5), and inhabits pine-oak forests from 2200–2360 m. a.s.l. *Phanaeus halffterorum* is considered a mycetophagous species (Edmonds 1979, 1994, Halffter and Edmonds 1982).

### Phanaeus bravoensis sp. n.

Taxon classificationAnimaliaColeopteraScarabaeidae

http://zoobank.org/41BF39B8-9D18-4275-9A1A-01BA830C3867

**Figure 6.**
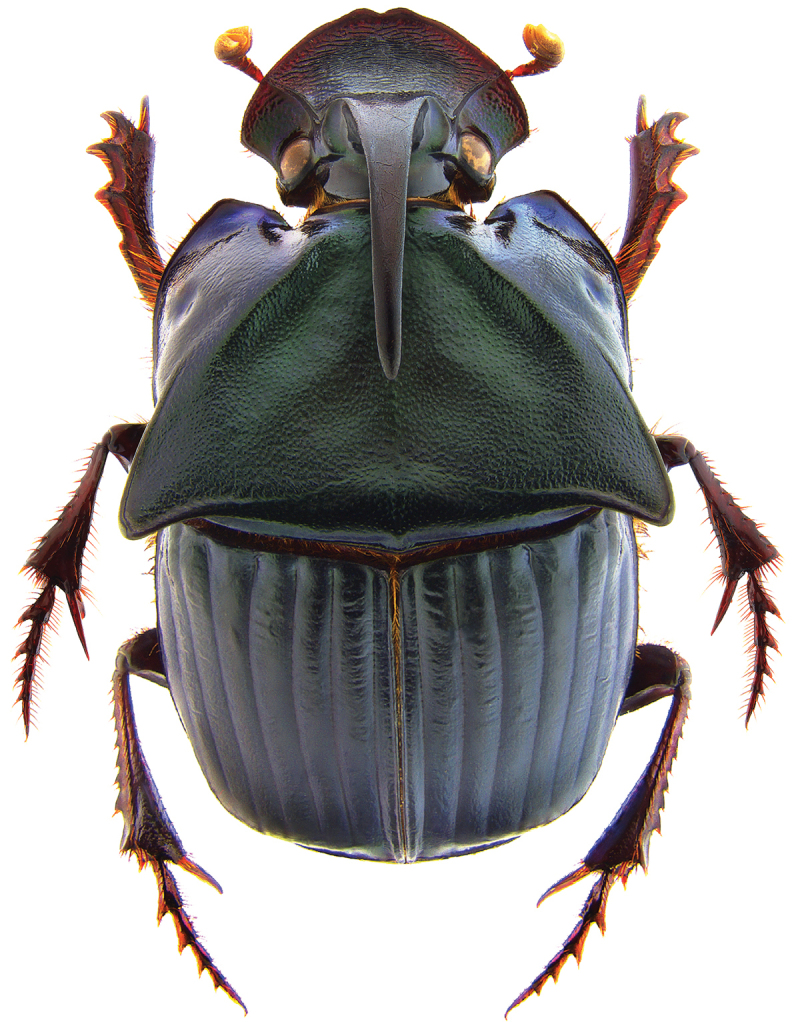
*Phanaeus bravoensis* sp. n. major male, dorsal view (holotype).

**Figure 7.**
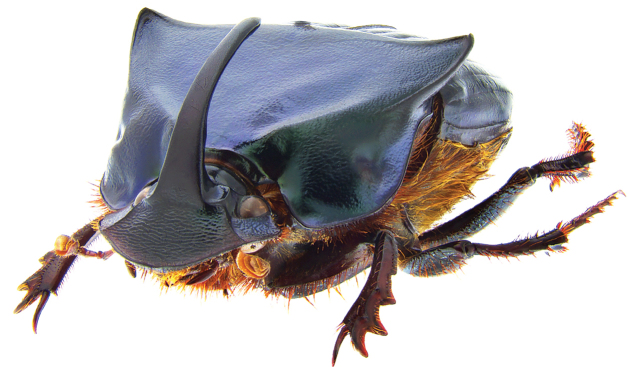
*Phanaeus bravoensis* sp. n. major male, lateral view (holotype).

**Figure 8.**
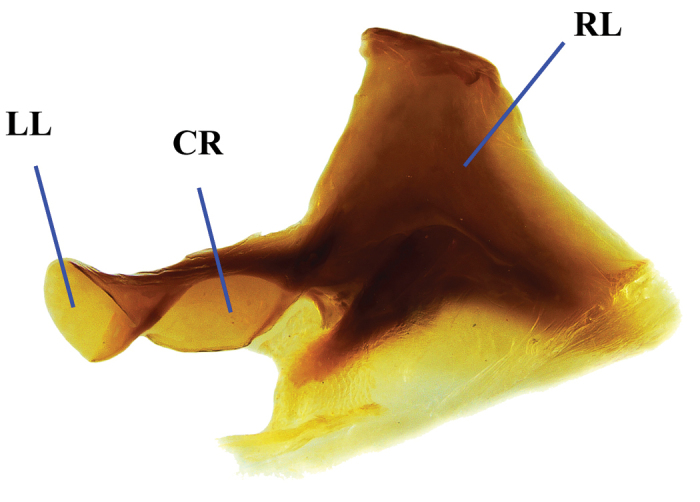
Lamella copulatrix of *Phanaeus bravoensis* sp. n. (holotype). Abbreviations: LL: left lobe, CR: central ridge, RL: right lobe.

**Figure 9.**
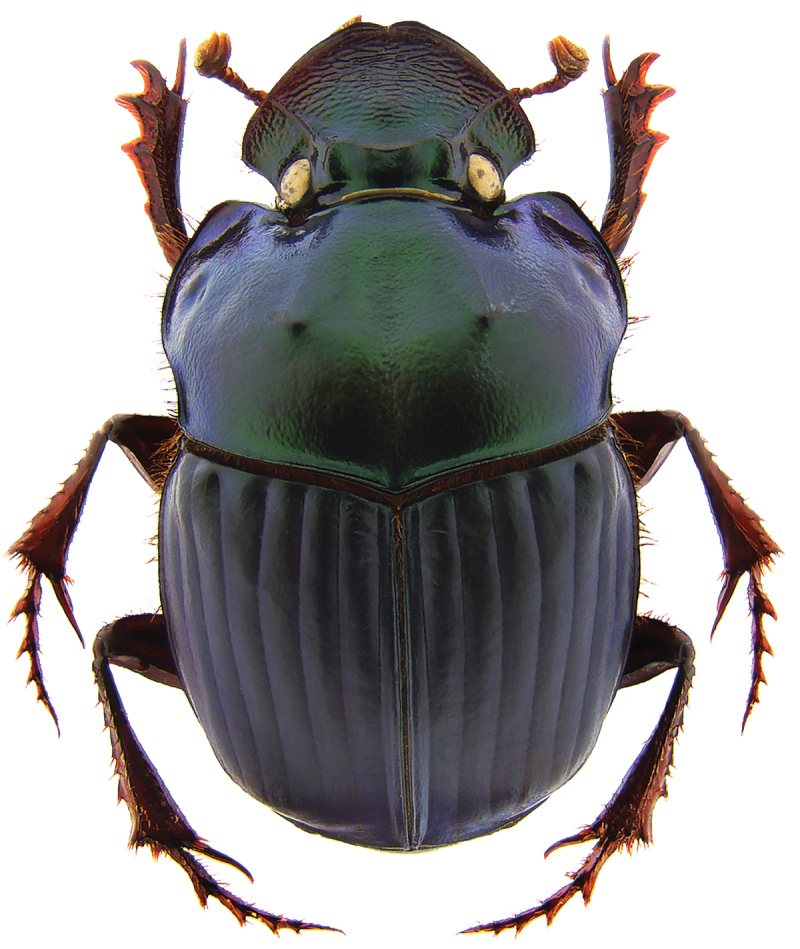
*Phanaeus bravoensis* sp. n. minor male, dorsal view (paratype).

**Figure 10.**
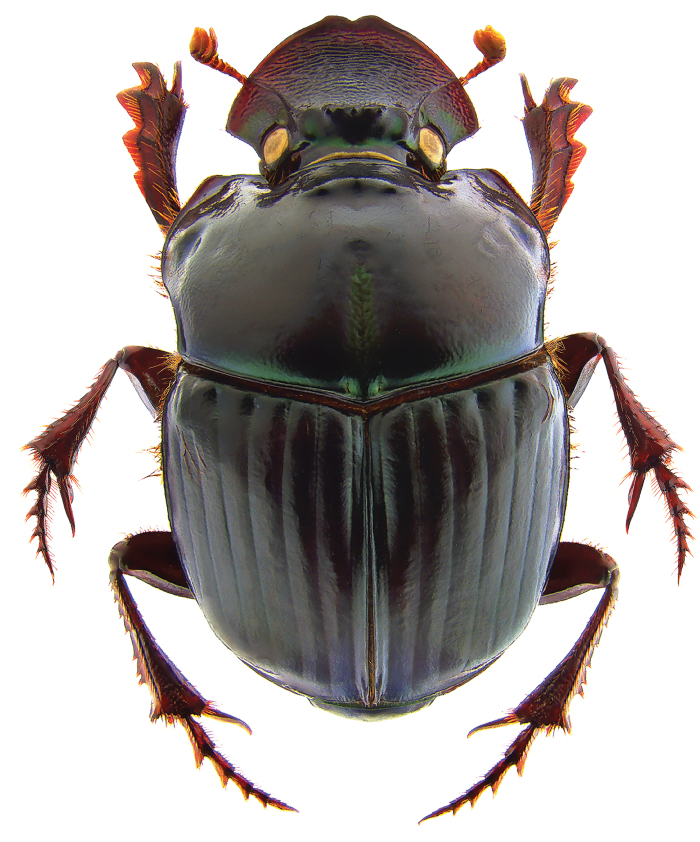
*Phanaeus bravoensis* sp. n. female, dorsal view (paratype).

Figs 6 , 7 , 8 , 9 , 10

Phanaeus halffterorum : Edmonds (1979: 99; *partim*), Arnaud (2002: 95–96), Edmonds (2003: 61, 65), Edmonds (2006: 31, 34, 36), Deloya et al. (2013: 90–92), Deloya et al. (2014: 77, 206), Edmonds (1994: 39–43, 101), Edmonds and Zídek (2012: 3, 5, 12, 52, 54), Moctezuma and Halffter (2017: 52, 54–55), Lizardo et al. (in press). Non halffterorum Edmonds, 1979.

#### Type material

(17 ♂♂, 13 ♀♀). Holotype major male pinned with genitalia in microvial (Figs 6–8): “MEXICO: Guerrero, Chilpancingo de los Bravo, entre Amojileca-Xocomanatlán, V-XI/2014, 17°33'41.17"N, 99°36'59.95"W, necrotrampa, bosque de encino-pino, 1860 m, Ernesto L. Huicochea col.”. Paratypes: 5 ♂♂, 1 ♀ same data as holotype; 5 ♂♂, 2 ♀♀ labeled “MEXICO: Guerrero, Chilpancingo, Amojileca, 434559 mE, 1941772 nM, 1772 msnm, bosque de *Quercus*-coníferas, 27/VI/2014, NTP, E. López-Huicochea Col.”; 2 ♂♂, 3 ♀♀ *ídem* except “03/XI/2014”; 4 ♀♀ *ídem* except “1860 msnm, 10/X/2014”; 2 ♂♂ labeled “MEXICO: Guerrero, Chilpancingo, Xocomanatlán, 432832 mE, 1938117 mN, 2100 msnm, bosque de coníferas-*Quercus*, 10/VIII/2014, NTP, E. López-Huicochea Col.”; 1 ♀ *ídem* except “04/IX/2014”; 1 ♂, 2♀♀ labeled “MEXICO: Guerrero, Chilpancingo, La Cimaroa, Ejido Amojileca, 431911 mE, 1939239 mN, 2150 msnm, bosque de Quercus magnolifolia, 10/VIII/2014, NTP, E. López-Huicochea Col.”; ”; 2 ♂♂ labeled “MEXICO: Guerrero, Chilpancingo, Acahuizotla, 28/06/2008 – 31/07/2008, veg. encino-pino, sitio 4, NTP IV, Madora Astudillo M col.”.

#### Type deposition.

Holotype 1 ♂ IEXA; paratypes: 1 ♂ TAMU; 1 ♂ JLSHC; 2 ♂♂, 1 ♀ VMC; 12 ♂♂, 12 ♀♀ temporally deposited in CDINECOL (12 paratypes will be permanently deposited in CNIN and 12 paratypes in FSCA).

#### Diagnosis.

Pronotal granulate sculpturing; major male lacks a tooth in the middle of anterior pronotal margin, pronotal triangle sides curved (Figs 6, 7); sutural margin of each elytron upturned to form a sharp ridge, which is progressively more elevated posteriorly and prolonged into a small, sharp tooth at apical angle; lamella copulatrix as in Fig. 8.

#### Description.

Holotype length 17.8 mm, width at bases of elytra 9.8 mm. **Head**: Clypeus black with metallic green bright, anterior margin weakly bidentate. Genae metallic green with granular rough sculpturing. Frons with a black cephalic horn, curved posteriorly over pronotum; lateral region of frons is metallic green and weakly rough with no evident punctures. **Pronotum**: Triangular pronotal disc with lateral fossae and dark metallic green coloring, flattened with lateral undulations. Lateral lines of pronotal disc recurved. Granulate sculpturing without punctuation. Posterior pronotal angles very salient, directed posterolaterally and slightly upturned apically. Basal fossae rounded but weakly impressed. **Elytra**: Striae fine, black colored, with small but well defined and regularly separated punctures, shagreened rough sculpturing. Interstriae of opaque appearance, dark metallic green coloring, weakly flattened, with shagreened sculpturing, small weakly impressed punctures and transverse roughness more evident on the first three interstriae. Sutural margin of each elytron forms a sharp ridge, which is elevated posteriorly and prolonged into a small tooth at the apical angle. **Pygidium**: Metallic dark green, glabrous, with weakly impressed small punctures and shagreened rough sculpturing. **Protibia**: Quadridentate with apical spur. **Aedeagus**: Similar to that of the *P. endymion* species group. Lamella copulatrix as Fig. 8.

#### Variation.

**Minor male (Fig. 9)**: Similar to major male, except the cephalic horn smaller or completely reduced to a frontal carina, posterolateral angles of pronotum reduced to small tubercles located on the central part of pronotum and lesser transverse roughness on the interstriae. The tooth on the elytra sutural margin is reduced. **Female (Fig. 10)**: As male except head black with green reflections in frons and genae, cephalic carina trituberculate with middle tubercle more elevated, pronotal sculpturing regularly reticular, most of pronotal disk dull black with a well-impressed coarse midline over posterior half of pronotum, pronotal process trituberculate, with the middle tubercle more elevated. **Size of paratypes.** Mean length 15.5 mm (13.3–17.1 mm), mean width 8 mm (6.8–9 mm).

#### Etymology.

Bravo + *ensis*. Bravo refers to type locality, Chilpancingo de los Bravo.

#### Remarks.

*Phanaeus bravoensis* is easily distinguished from the closely related *P. halffterorum* by geographic distribution and morphological characters: *P. bravoensis* major male lacks a tooth in the middle of anterior pronotal margin and its pronotal triangle sides are curved (Figs 6, 7), whereas these are straight on *P. halffterorum* (Figs 1, 2). Differences are observed between the lamella copulatrix (more developed left lobe in *P. halffterorum*, strongly developed central ridge and right lobe in *P. bravoensis*; Figs 3, 8) and shape of female pronotum. In *P. bravoensis* mayor female pronotal midline is stronger impressed; pronotal tubercles are located in similar position and of equal size in *P. halffterorum*, while pronotal central tubercle is in anterior position and bigger than lateral tubercles in *P. bravoensis* (Figs 4, 10).

#### Type locality.

MEXICO, Guerrero, Chilpancingo de los Bravo.

#### Distribution and Ecology.

This species occurs in the Sierra Madre del Sur, Guerrero (Fig. 5), in coniferous-oak forest between 750–2150 m. a.s.l. Specimens have been collected with carrion and dung baited pitfall traps, and attracted to light. Therefore, *P. bravoensis* seems to be copronecrophagous (Edmonds 1994, Deloya et al. 2013, 2014).

### Phanaeus huichol sp. n.

Taxon classificationAnimaliaColeopteraScarabaeidae

http://zoobank.org/EAAACC06-DAD3-4BB3-8790-97A5F96E20F5

**Figure 11.**
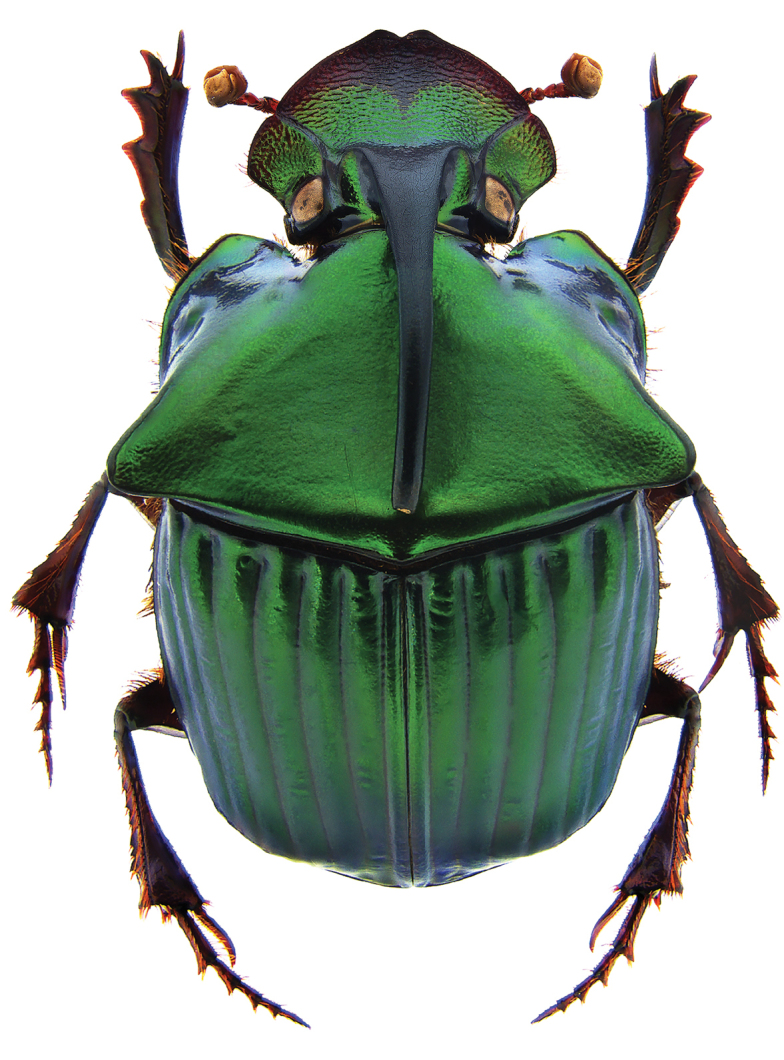
*Phanaeus huichol* sp. n. major male, dorsal view (holotype).

**Figure 12.**
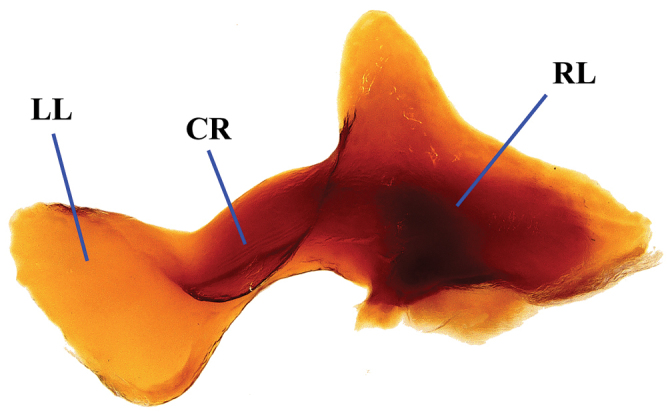
Lamella copulatrix of *Phanaeus huichol* sp. n. (holotype). Abbreviations: LL: left lobe, CR: central ridge, RL: right lobe.

**Figure 13.**
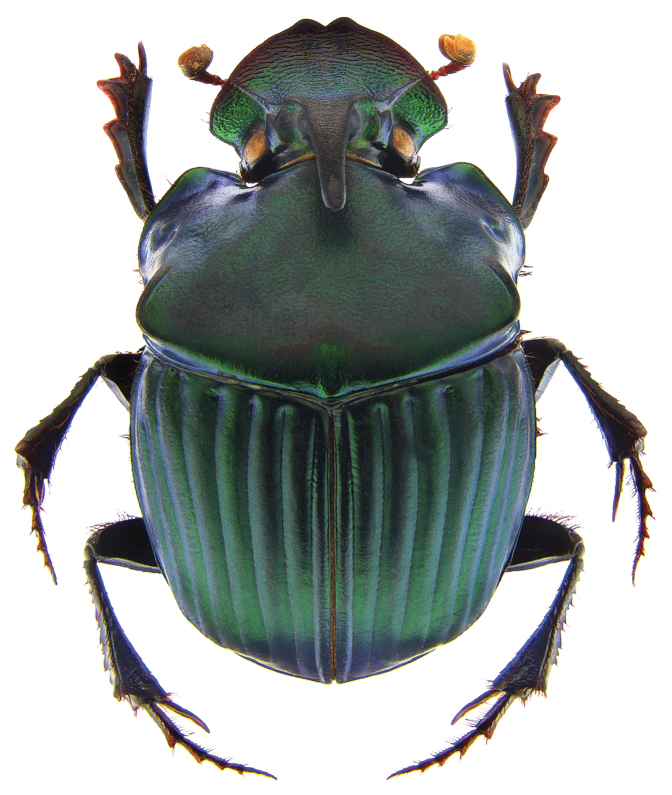
*Phanaeus huichol* sp. n. minor male, dorsal view (paratype).

**Figure 14.**
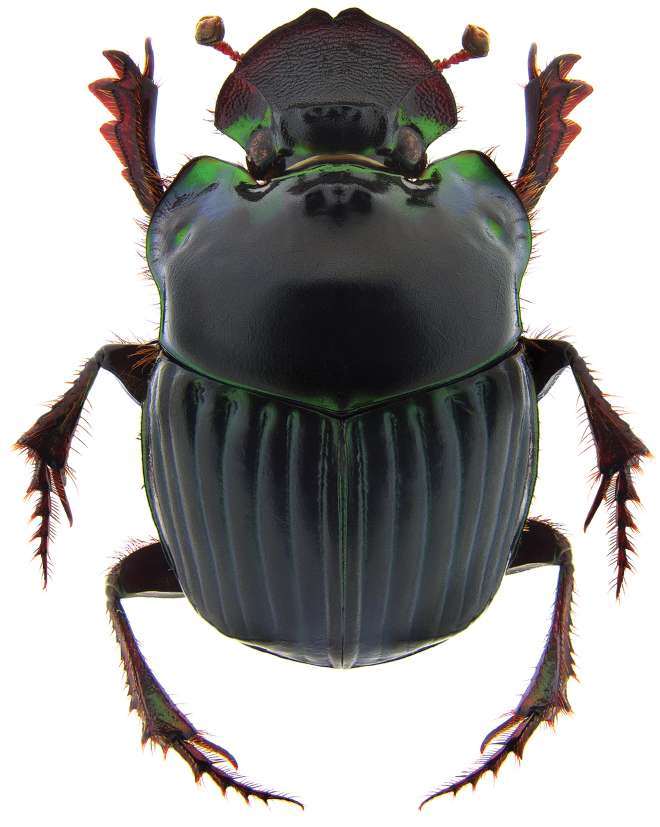
*Phanaeus huichol* sp. n. female, dorsal view (paratype).

**Figure 15.**
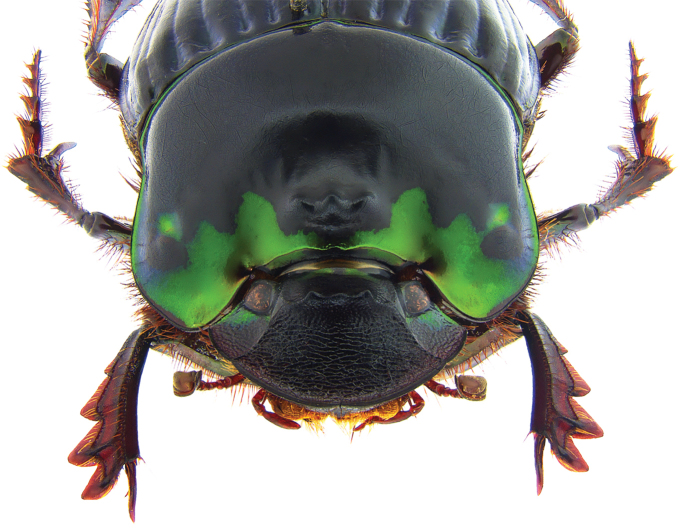
*Phanaeus huichol* sp. n. female, frontal view (paratype).

**Figure 16.**
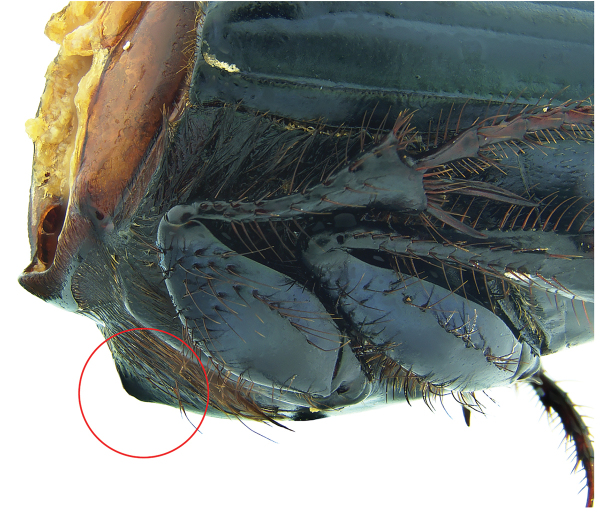
*Phanaeus huichol* sp. n. anterior metasternal angle pointed out with a red circle, lateral view (paratype).

Figs 11 , 12 , 13 , 14 , 15 , 16 , 18

Phanaeus endymion : Edmonds (1994: 39–44, 101; *partim*), Arnaud (2002: 94–95), Edmonds (2003: 61, 65), Quiroz-Rocha et al. (2008: 29, 33, 34, 36, 37), Edmonds and Zídek (2012: 1, 3, 5-8, 12, 13, 52, 53), Moctezuma and Halffter (2017: 47, 52-55), Lizardo et al. (in press). Non endymion Harold, 1863.

#### Type material

(12 ♂♂, 8 ♀♀). Holotype major male pinned with genitalia in microvial (Figs 11, 12): “MEXICO: Jalisco, 3 mi. NE Mazamitla, VII-12-1982, in moist cow dung, Fred G. Andrews col.” Paratypes: 1 ♂, 1 ♀ labeled “MEXICO, Jalisco, Sierra de Talpa (CT), 1470 m, 11/13-VIII-2010, Nogueira col.”; 2 ♂♂ labeled “MEXICO, Jalisco, Mpio. Talpa de Allende, 20°13'03.4"N, 104°45'58.8"W, 1655 m, 18–22.vii.09, fungi, WD Edmonds & P. Reyes cols.”; 1 ♂, 1 ♀ labeled “MEXICO, Jalisco, S. Manantlán, 1650 m, 18-20/VII/95, G. Nogueira col.”; 1 ♂, 1 ♀ labeled “MEXICO, Jalisco, Mixtlán, 1758 m, 13-VIII-2012, G. Nogueira col.”; 1 ♂, 1 ♀ labeled “JA: hwy 200, 21 mi S Puerto Vallarta, vii-9,10-84, 2310’, ex fungi, S McCleve, P. Jump cols.”; 1 ♀ labeled “MEX, Jal., 4200’, 10mi SW Autlán, IX.19.71, A. Newton col.”; 1 ♂ labeled “MEXICO: Jalisco, Autlán, Puerto los Mazos, Bosque de galería, 1580m, Necrotrampa, x.2000, H. Bustos col”; 1 ♀ *ídem* except “Encino caducifolio, 1480m, xi.2000”; 1 ♂ labeled “MEXICO: Jalisco, Zapopan, Los Guayaboa (La Guayaba?), BEpert, 1600m, 8.vii.1995, NTP-80, G. Quiroz y J.L. Navarrete cols.”; 1 ♀ *ídem* except “8.vii-4.viii.1994”; 1 ♀ *ídem* except “14.ix-15.x.1994, pulpo, G. Quiroz y J.L. Navarrete cols.”; 1 ♂ labeled “MEXICO: Nayarit, Sierra el Nayar, July 1994, Guillermo Nogueira lgt.”; 1 ♂ labeled “MEXICO, Nayarit, Sierra del Nayar, Chapalilla, 1280 m, 17-VII-94, coprotr. G. Nogueira col.”; 1 ♂ *ídem* except “17.xii.1994”.

#### Type deposition.

Holotype 1 ♂ and six paratypes (4 ♂♂, 2 ♀♀) TAMU; rest of paratypes as follows: 1 ♂, 1 ♀ MXAL; 2 ♂♂, 1 ♀ VMC; 1 ♂, 1 ♀ JLSHC, 3 ♂♂, 3 ♀♀ CEMT.

#### Diagnosis.

Dorsum metallic green; anterior margin of pronotum projected upwards; acute posterolateral angles of pronotum (Fig. 11); anterior metasternal angle almost right angled but with rounded apex in lateral view (Fig. 16); lateral metasternal angles evanescent (Fig. 18); lamella copulatrix as in Fig. 12.

#### Description.

Holotype length 17.6 mm, width at base of elytra 9.8 mm. **Head**: Clypeus dark brown with bright metallic green, anterior margin bidentate. Genae metallic green with granular sculpturing. Frons with a black cephalic horn, curved posteriorly over pronotum; lateral region of frons is metallic green weakly rough with coarse and weakly impressed punctures. **Pronotum**: Triangular pronotal disc with lateral fossae and metallic olive green coloring, flattened with lateral undulations. Lateral lines of pronotal disc not impressed. Sculpturing shagreened without punctures. Anterior pronotal angles wide and rounded. Posterior pronotal angles salient, directed laterally and weakly upturned apically. Basal fossae weakly impressed. Two lateral carinas are impressed near to pronotal apex. **Elytra**: Striae black-green colored, shagreened with small well-spaced punctures and weakly rough sculpturing. Interstriae strongly impressed with small punctures. **Pygidium**: Metallic olive green, with well-impressed punctures and shagreened rough sculpturing. **Protibia**: Quadridentate with apical spur. **Aedeagus**: Similar to that of *P. endymion* species group. Lamella copulatrix as Fig. 12.

#### Variation.

This species shows variation in color, being olive green or dark green with blue reflections. **Minor male (Fig. 13)**: Similar to major male, except the cephalic horn smaller and the posterolateral angles of pronotum reduced. **Female (Figs 14, 15): **As male except head black with green reflections in frons and genae, cephalic carina trituberculate, pronotal sculpturing shagreened, most of pronotal disk dull black, pronotal process trituberculate. **Size of paratypes.** Mean length 17.5 mm (16–18.7 mm), mean width 9.4 mm (8.7–9.8 mm).

#### Etymology.

The name of the new species refers to the Huichol ethnic group, which inhabits part of the geographic region where the type series was collected.

#### Remarks.

**Figure 17.**
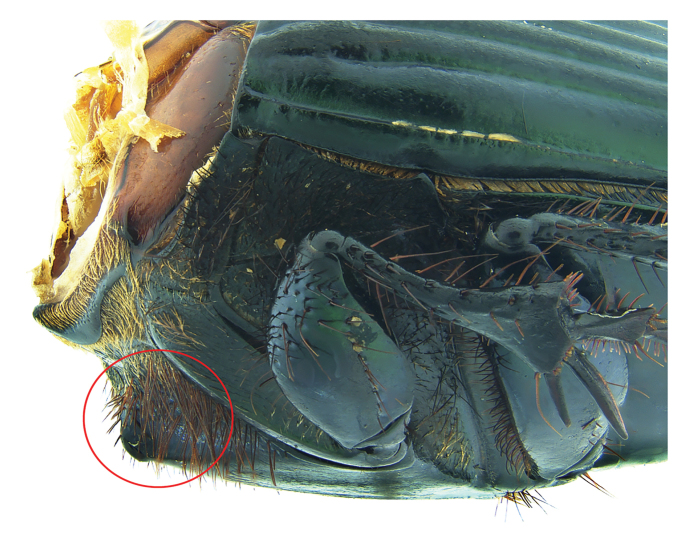
*Phanaeus zoque* anterior metasternal angle pointed out with a red circle, lateral view (paratype).

**Figure 18.**
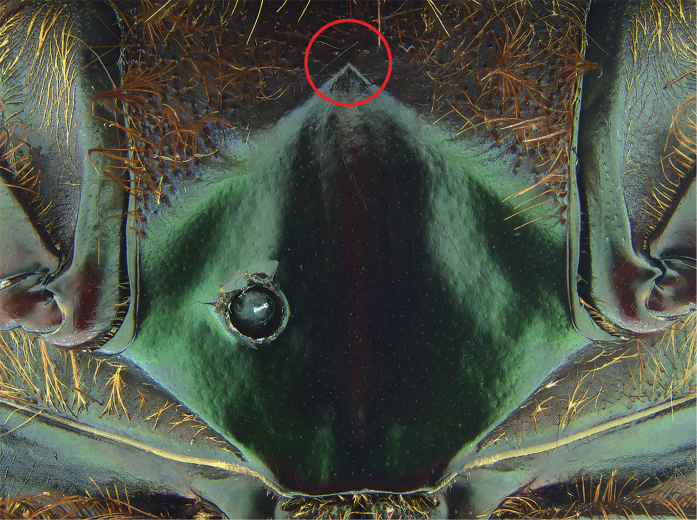
*Phanaeus huichol* sp. n. metasternum (paratype). The anterior metasternal angle is indicated with a red circle.

**Figure 19.**
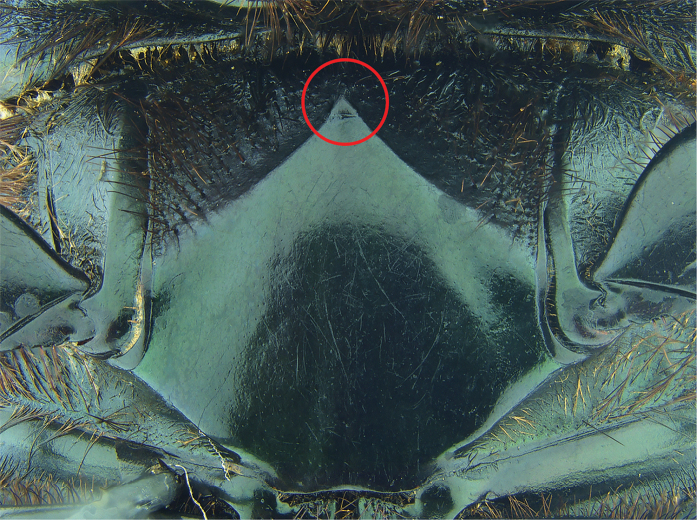
*Phanaeus zoque* metasternum (paratype). The anterior metasternal angle is indicated with a red circle.

*Phanaeus huichol* is easily distinguished from the closely related *P. zoque* by its geographic distribution and morphological characters: *Phanaeus huichol* male shows two elongate and weak tumescences near to pronotal apex, while *P. zoque* presents two strong tubercles; the anterior lateral angles of the pronotum of *P. huichol* are wider and more rounded than those of *P. zoque*. Major females of *P. huichol* show tridentate pronotal projections with teeth of similar size (Fig. 15), while in the *P. zoque* major female the middle dent resembles a carina, with smaller lateral teeth. In both males and females of *P. huichol* the anterior metasternal angle is obtuse in lateral view (Fig. 16), whereas in *P. zoque* this is almost right-angled but with a rounded apex (Fig. 17). Anterior metasternal angles are notably less angular in *P. huichol* (Fig. 18), while they are more evident in *P. zoque* (Fig. 19). Differences in angulation are also evident in other ventral sternites (lateral mesometasternal angles evanescent in *P. huichol*, those angles well defined and slightly curved in *P. zoque*). *Phanaeus huichol* is restricted to the Pacific slope of Mexico (Jalisco and Nayarit), while *P. zoque* is found in the Mexican southeast (Oaxaca and Chiapas).

#### Type locality.

MEXICO, Jalisco, Mazamitla.

#### Distribution and Ecology.

This species occurs in the Sierra Occidental of Jalisco and the Sierra del Nayar (Nayarit), in coniferous-oak forests, cloud forests and riparian forests, between 700–1760 m a.s.l. This species seems to be generalist, since specimens have been collected with carrion, dung and fungus.

### Key to the *Phanaeus endymion* species group (modified from Edmonds and Zídek 2012 and Moctezuma and Halffter 2017).

**Table:** 

1	Sutural margin of each elytron upturned to form a sharp ridge, which is progressively more elevated posteriorly and prolonged into a small, sharp tooth at apical angle; elytral margin slightly excised adjacent to this tooth	**2**
–	Sutural margin of elytra simple. Color and distribution variable	**3**
2	Major male with a tooth in the middle of anterior pronotal margin, pronotal triangle sides straight (Figs 1, 2). Lamella copulatrix as in Fig. 3. South-western Mexico State (Estado de México)	***P. halffterorum***
–	Major male lacks a tooth in the middle of anterior pronotal margin, pronotal triangle sides curved (Figs 6, 7). Lamella copulatrix as in Fig. 8. Sierra Madre del Sur of central Guerrero	***P. bravoensis* sp. n.**
3	Triangular pronotal disk of male evenly and densely but finely granulate (×10), granules in most specimens larger and becoming squamose along lateral margins of disk and extending onto posterolateral angles (when distinctly developed); sides of pronotum roughened (×10), lacking distinct punctures except behind lateral fossae. Female pronotum minutely roughened, evenly, distinctly punctate (×10), punctures becoming smaller dorsally but not disappearing altogether; disk impressed medially as a distinct furrow visible to unaided eye, extending forward from posterior margin to near middle of disk. Puebla-Oaxaca Mountain System and Sierra Madre del Sur of south-central Oaxaca	***P. zapotecus***
–	Pronotal disk of male either lacking distinct granulation, or, if granules present, these are minute and restricted along lateral margins of disk; sides of pronotum smooth, minutely punctate. Female pronotum (fig. 140) smooth, punctures (×50) fine and usually restricted to sides; median furrow lacking or at most indicated by fine, scarcely visible line	**4**
4	Elytral interstriae distinctly flattened and uniformly dull (more convex and shiny in some Central American populations); striae not strongly impressed basally, anterior ends in most specimens bearing deep punctures rather than large fossae. Male: Pronotal disk dull, velvety smooth medially, finely asperate, brighter laterally. Female: Pronotum evenly convex, lacking anteromedial concavity even in largest specimens, bearing three round, smooth tubercles in transverse line near anterior margin. Head and pronotum largely highly shiny metallic red to nearly completely dull black with metallic red restricted to ridges and isolated areas on anterior part of pronotum; elytra dull to weakly shiny black; pygidium usually metallic red medially, green peripherally, in some completely red or green. Southern Nicaragua through Central America into western Colombia and Ecuador	***P. pyrois***
–	Elytral interstriae evenly convex and glossy midlongitudinally; striae impressed basally as distinct fossae. Male: Pronotal disk velvety smooth medially, finely aspirate laterally and sometimes also medially. Female: Pronotum with anteromedial concavity bounded anteriorly by a raised U- or V-shaped ridge	**5**
5	Dorsum dark blue or shiny green; in few specimens shiny green with strong yellow reflections. Anterior margin of pronotum projected forwards. Relatively rounded posterolateral angles of pronotum. Internal sack of aedeagus and lamella copulatrix as in Moctezuma and Halffter (2017; Fig. 16). Southwestern Mexico to Honduras	***P. endymion***
–	Dorsum metallic green. Anterior margin of pronotum projected upwards. Acute posterolateral angles of pronotum	**6**
6	Anterior metasternal angle obtuse in lateral view (Fig. 17). Lateral metasternal angles well defined and slightly curved (Fig. 19). Few specimens olive green with golden/reddish reflections. Internal sack of aedeagus and lamella copulatrix as in Moctezuma and Halffter (2017; Fig. 15). Eastern Oaxaca and western Chiapas	***P. zoque***
–	Anterior metasternal angle almost right angled but with rounded apex in lateral view (Fig. 16). Lateral metasternal angles evanescent (Fig. 18). Lamella copulatrix as in Fig. 12. Jalisco and Nayarit	***P. huichol* sp. n.**

## Discussion

Notwithstanding the fact that a review and new key are required for the *endymion* species group, we have found no reason to delay publication of this new species. We do not include a new key to separate the *endymion* species group. As an alternative, however, we modified the keys presented by former studies (Edmonds and Zídek 2012, Moctezuma and Halffter 2017). We also considered species distribution, ecology, and trophic preferences as additional criteria to separate the new species.

*Phanaeus bravoensis* and *P. halffterorum* exhibit unique combinations of character states in the external morphology and in the sclerites of the internal sack of the aedeagus, and these character combinations are sufficient to consider them as distinct species (Wheeler and Platnick 2000). *Phanaeus bravoensis* and *P. halffterorum* seem to be closely related species, because of similarity in the granular pronotal microsculpture, pronotum shape and the apical tooth in the base of the elytra.

*Phanaeus bravoensis* and *P. halffterorum* occupy distinct ecological niches. They both inhabit coniferous-oak forests, but *P. halffterorum* is a mycetophagous specialist while *P. bravoensis* is attracted to dung and carrion (Edmonds 1979, 1994, 2003, Deloya et al. 2014). Both species are geographically isolated and endemic to small montane areas. *Phanaeus bravoensis* is restricted to the Sierra Madre del Sur (750-2150 m. a.s.l.) in the surroundings of Chilpancingo de Los Bravo, while *P. halffterorum* has been reported from the mid highlands of the central region of the Trans-Mexican Volcanic Belt (2200-2360 m. a.s.l.), in the surroundings of Temascaltepec. Arnaud (2002) mentioned that *P. halffterorum* is located in the “Federal District”, but this location is doubtful and the name could have been confused with State of Mexico (Estado de México). Climatic conditions where these species are found differ, being tropical or sub-tropical for *P. bravoensis* and temperate for *P. halffterorum*. Lizardo et al. (in press) states that potential distribution modeling of species could not be performed using localities of *P. halffterorum* from the State of Mexico and Guerrero simultaneously, probably because of the lack of information and/or taxonomical/geographical errors. We agree with the view of Lizardo et al. (in press) and have therefore included additional localities and corrected the taxonomical issue that involved *P. halffterorum*.

Vicariance is likely the process that led the radiation of *P. bravoensis* and *P. halffterorum*. Aridification of the Balsas Valley during the Pleistocene could have isolated the populations of a common ancestor (Edmonds 1994). This hypothesis considers a relatively recent origin of this species within the *endymion* species group. We consider the possibility that *P. bravoensis*, *P. halffterorum* and *P. zapotecus* represent a phyletic line within the *endymion* species group, characterized by the presence of a granular pronotal microsculpture. Molecular phylogenetic studies are required to confirm or refute our hypothesis on the vicariant origin of *P. bravoensis*, the geological period of divergence and the existence of the lineage *bravoensis-halffterorum-zapotecus*.

*Phanaeus huichol* has been traditionally confused with *P. endymion*, a noteworthy fact considering the number of important reviews that have been published in recent years (Edmonds 1994, Arnaud 2002, Edmonds and Zídek 2012). Species potential distribution modeling clearly demonstrates a geographical segregation among *P. huichol*, *P. endymion* and *P. zoque* (Lizardo et al. in press). This new species seems to be closely related to the recently described *P. zoque* in size, coloring and habitat preference, but there are clear differences in external and genital morphology. On the other hand, an important disjunction exists between the distributions of both species: they are separated by ≈900 km of distance, including the presence of important biogeographic barriers (i.e., the Tehuantepec Isthmus, the Sierra Madre del Sur and the Balsas Valley). *Phanaeus huichol* could represent a relatively ancient colonization by the *P. endymion* species group in the Central Pacific region of Mexico, that drove a later isolation of this species in the westernmost areas of the Trans-Mexican Volcanic Belt.

While both species prefer pine-oak forests, *P. huichol* also inhabits cloud forests, while *P. zoque* has not been collected despite intensive sampling effort in cloud forests of Los Chimalapas (Moctezuma and Halffter 2017). Trophic habits of *P. huichol* are well known, being a generalist species attracted to different kinds of dung, carrion, and fungus, while *P. zoque* has been collected only on dung (Quiroz-Rocha et al. 2008, Moctezuma and Halffter 2017). Feeding habits of *P. huichol* could be related to xeric conditions during dry season, when dung becomes an extremely ephemeral resource that rapidly loses humidity and dietary quality, and additional food sources are required (Halffter and Edmonds 1982, Moctezuma et al. 2016). Elevation tolerance of these species seems to overlap and they are endemic to montane areas, although the altitudinal range of *P. huichol* is greater: *P. huichol* is found between 700-1760 m. a.s.l., while *P. zoque* inhabits between 918-1325 m. a.s.l.

## Supplementary Material

XML Treatment for Phanaeus halffterorum

XML Treatment for Phanaeus bravoensis

XML Treatment for Phanaeus huichol

## Supplementary materials

Supplementary material 1 Table of localities Data type: occurrence This dataset is made available under the Open Database License (http://opendatacommons.org/licenses/odbl/1.0/). The Open Database License (ODbL) is a license agreement intended to allow users to freely share, modify, and use this Dataset while maintaining this same freedom for others, provided that the original source and author(s) are credited.Victor Moctezuma, José Luis Sánchez-Huerta, Gonzalo Halffter
